# Citrinin Monomer and Dimer Derivatives with Antibacterial and Cytotoxic Activities Isolated from the Deep Sea-Derived Fungus *Penicillium citrinum* NLG-S01-P1

**DOI:** 10.3390/md17010046

**Published:** 2019-01-10

**Authors:** Weiyi Wang, Yanyan Liao, Beibei Zhang, Maolin Gao, Wenqian Ke, Fang Li, Zongze Shao

**Affiliations:** 1 Key Laboratory of Marine Genetic Resources, State Key Laboratory Breeding Base of Marine Genetic Resources, Fujian Key Laboratory of Marine Genetic Resources, Fujian Collaborative Innovation Centre for Exploitation and Utilization of Marine Biological Resources, Third Institute of Oceanography, Ministry of Natural Resources, Xiamen 361005, China; zbb953372968@163.com (B.Z.); g17785208284@163.com (M.G.); 15260587285@163.com (W.K.); lifang@tio.org.cn (F.L.); shaozongze@tio.org.cn (Z.S.); 2 Key Laboratory of Urban Environment and Health, Institute of Urban Environment, Chinese Academy of Sciences, Xiamen 361021, China; yyliao@iue.ac.cn

**Keywords:** *Penicillium*, citrinin dimer, antibacterial, cytotoxic

## Abstract

Two previously unreported citrinin dimer derivatives, penicitol D (**1**) and 1-*epi*-citrinin H1 (**2**), were isolated from the culture of a deep sea-derived fungus *Penicillium citrinum* NLG-S01-P1, together with 11 biogenetic related compounds (**3**–**13**). A plausible biogenetic pathway for compounds **2**–**4** was proposed. Their structures, including absolute configurations, were established through analysis of extensive spectroscopic data and time-dependent density functional theory (TD-DFT) ECD calculations. Compounds **1** and **2** showed antibacterial activities against methicillin-resistant *Staphylococcus aureus* (MRSA). Compounds **5** and **10** displayed relatively stronger activities than the other compounds against *Vibrio vulnificus* and *Vibrio campbellii*. Compound **1** showed the most potent cytotoxic activity towards the HeLa cell.

## 1. Introduction

**Figure 1:**
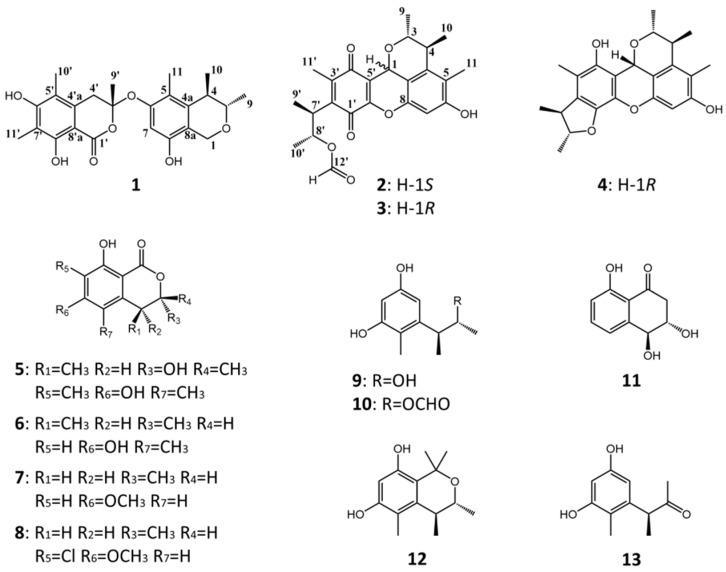
Structure of compounds **1**–**13**.

Citrinins are a family of fungal mycotoxins characterized by colored crystals and conjugated bonds in their chemical structures. Citrinin was first isolated from a species of *Penicillium citrinum* and is also produced by the *Monascus* and *Aspergillus* genera. Biosynthetic studies of citrinins have been carried out using a variety of isotopic labeling strategies [1]. Citrinin is well known as a polyketide mycotoxin found in stored grains and grain-based products with different types of toxicity—including nephrotoxicity, genotoxicity, and carcinogenicity. As such, several studies have been carried out for the detoxification of citrinin which demonstrated that its decomposition coincided with a decrease in cytotoxicity in most cases. Phenol A (**9**) and citrinin H2 (**10**) were decomposition products of citrinin under high temperature [2,3,4]. However, citrinin H1, as a dimeric product formed upon heating at 140 °C in the presence of water, contributed to an increase in cytotoxicity [5]. Despite its toxic properties, there is increasing evidence that supports the existence of other biological activities including anticancer, antibacterial, antifungal, and neuro-protective effects in vitro [6]. During our search for bioactive metabolites from marine-derived microorganisms, a *P. citrinum* strain NLG-S01-P1—isolated from a seawater sample at a depth of 4650 m (20°09′07.6067″ N, 160°15′07.6355″ E) in the West Pacific Ocean in 2017—attracted our attention. Studies on bioactive constituents of the ethyl acetate (EtOAc) extract led to the isolation of two previously unreported citrinin dimer derivatives, penicitol D (**1**) and 1-*epi*-citrinin H1 (**2**), together with 11 biogenetic related compounds (**3**–**13**): citrinin H1 (**3**); penicitrinol A (**4**); (3*S*,4*S*)-sclerotinin A (**5**); stoloniferol B (**6**); (3*R*)-6-methoxymellein (**7**); (3*R*)-6-methoxy-7-chloromellein (**8**); phenol A (**9**); citrinin H2 (**10**); (3*S)*-hydroxy-4-*epi*-isosclerone (**11**); (3*R*,4*S*)-6,8-dihydroxy-1,1-dimethyl-3,4,5-trimethylisochroman (**12**); and (3*S*)-(3’,5’-dihydroxy-2’-methylphenyl)-2-butanone (**13**) (Figure 1). Their structures were elucidated through spectroscopic methods, and both their cytotoxic and antibacterial activities were evaluated.

## 2. Results and Discussion

**Figure 2:**
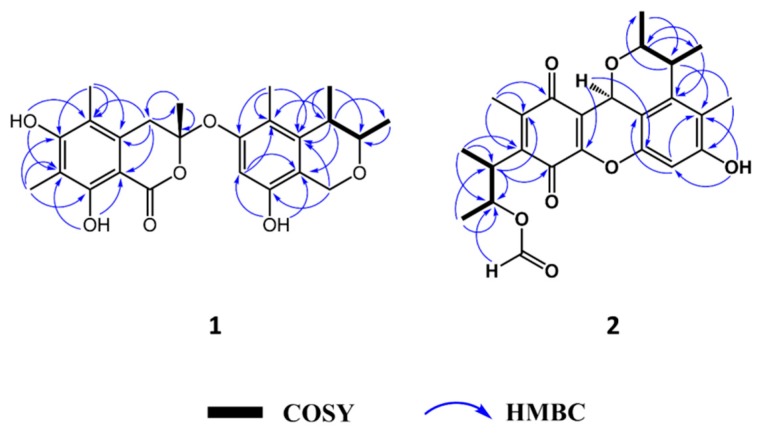
Key COSY and HMBC correlations of compounds **1** and **2**.

**Figure 3:**
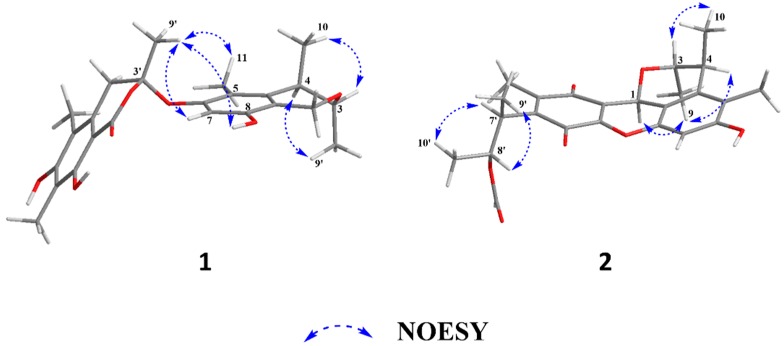
Key NOESY correlations of compounds **1** and **2**.

**Figure 4:**
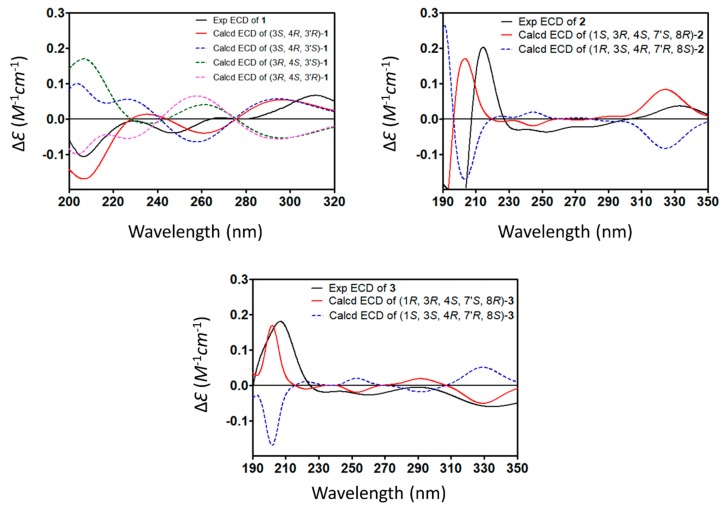
Calculated and experimental ECD of compounds **1**–**3**.

Penicitol D (**1**) was obtained as a yellow amorphous solid. Its molecular formula was established as C_24_H_28_O_7_ by high-resolution electrospray ionization mass spectroscopy (HR-ESI-MS) (*m*/*z* 427.1747 [M − H]^−^; calcd. for C_24_H_27_O_7_, 427.1757; ∆—2.3 ppm), implying 11 degrees of unsaturation (Figure S5). The ^13^C/DEPT and HSQC spectra revealed the presence of six methyl groups (C-9, C-10, C-11, C-9′, C-10′, and C-11′), two methylene groups (C-1 and C-4′), two sp^3^-hybridized methine groups (C-3 and C-4) including one oxygen-bearing carbon (C-3), one sp^2^-hybridized methine group (C-7), one sp^3^-hybridized quaternary oxygen-bearing carbon (C-3′), as well as 12 sp^2^-hybridized quaternary carbons (C-4a, C-5, C-6, C-8, C-8a, C-1′, C-4′a, C-5′, C-6′, C-7′, C-8′ and C-8′a) including three oxygen-bearing carbon (C-6, C-6′ and C-8′) and one carbonyl carbon (C-1′), which partially suggested that penicitol D (**1**) was composed of two aromatic ring systems derived from a citrinin molecule (Figures S3 and S6). The ^1^H-^1^H COSY spectrum of **1** showed the correlations of a methyl (H-9) with an oxygen-bearing methine (H-3), H-3 with H-4, and H-4 with another methyl (H-10), indicating the existence of a 2-oxybutane unit in one aromatic ring moiety (Figure 2 and Figure S7). The HMBC correlations of H-1 to C-4a/C-8, H-4 to C-5/C-8a, H-11 to C-6/C-4a, H-7 to C-5/C-8a, and OH-8 to C-7/C-8a allowed the establishment of a 3,4-dimethyl-5-methyl-8-hydroxy isochroman unit. The remaining NMR signals accounted for the assignment of a penta-substituted isochromanone moiety, which was in part supported by the HMBC correlations from H-4′ to C-4′a/C-8′a/C-5′, H-9′ to C-4′/C-1′, H-10′ to C-4′a/C-6′, OH-6′ to C-5′/C-7′, H-11′ to C-6′/C-8′, and OH-8′ to C-7′/C-8′a (Figure 2 and Figure S4). The planar structure of **1** was constructed by connecting the isochromanone and isochroman units via an oxygen atom by the chemical shifts of C-3′ (δ*_C_* 102.3) and C-6 (δ*_C_* 154.2) combined with NOESY correlations of H-9′ with H-7, OH-8, and H-11 (Figure 3 and Figure S8). The *anti*-configuration of C-3 and C-4 in **1** was determined by the small coupling constant ^3^*J*_3,4_ (2.3) (Table 1), which was also confirmed by the NOESY correlations of H-4 with H-9 and H-3 with H-10 (Figure 3). To unambiguously determine the absolute configuration of compound **2**, four possible isomers, (3*S*, 4*R*, 3′*R*)-**1** and (3*S*, 4*R*, 3′*S*)-**1** as well as their corresponding mirror images (3*R*, 4*S*, 3′*S*)-**1** and (3*R*, 4*S*, 3′*R*)-**1**, were proposed, and their ECD spectra were calculated by time-dependent density functional theory (TD-DFT) (Tables S1 and S2). The experimental ECD spectrum of **1** was in good agreement with the calculated ECD spectrum of (3*S*, 4*R*, 3′*R*)-**1** (Figure 4). Thus, the absolute configuration at C-3, C-4, and C-3′ of **1** was established as *S*, *R* and *R*, respectively, and the structure of **1** is shown in Figure 1.

**Scheme 1:**
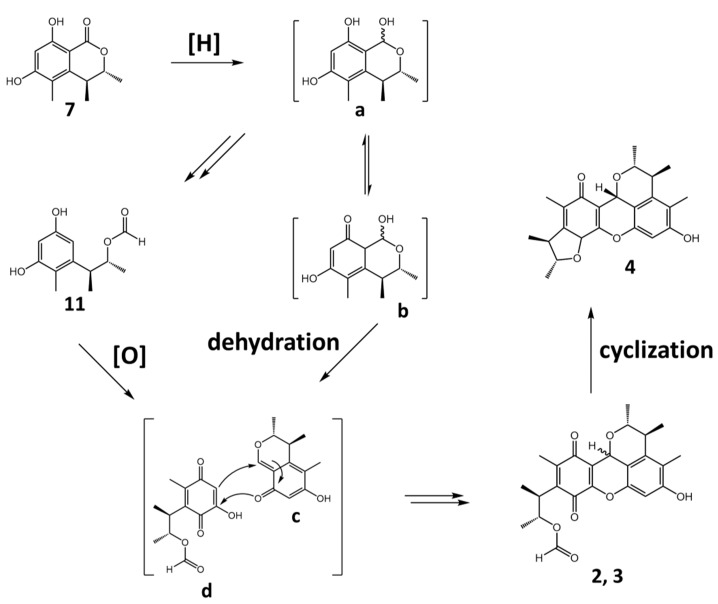
Postulated dimeric mechanism of **2**–**4**.

1-*epi*-citrinin H1 (**2**) was obtained as a red amorphous solid. Its molecular formula was elucidated as C_24_H_26_O_7_ by HR-ESI-MS (*m*/*z* 449.1576 [M + Na]^+^; calcd. for C_24_H_26_O_7_Na, 449.1576; ∆—0.0 ppm), implying 12 degrees of unsaturation (Figure S13). The ^13^C/DEPT and HSQC spectra showed the existence of six methyl groups (C-9, C-10, C-11, C-9′, C-10′, and C-13′), five sp^3^-hybridized methine groups (C-1, C-3, C-4, C-7′, and C-8′) including three oxygen-bearing carbons (C-1, C-3, and C-8′), two sp^2^-hybridized methine units (C-7 and C-12′), and 11 sp^2^-hybridized quaternary carbons (C-1′, C-2′, C-3′, C-4′, C-5′, C-6′, C-5, C-6, C-8, C-4a, and C-8a) including two carbonyl carbons (C-1′ and C4′) and three oxygen-bearing carbons (C-6′, C-8, and C-6) (Figures S11 and S14). A detailed analysis of ^1^H NMR, ^13^C NMR, ^1^H-^1^H COSY and HMBC techniques revealed that 1-*epi*-citrinin H1 (**2**) shared the same planar structure as citrinin H1 (**3**) with different stereochemical properties (Figures S12 and S15). NOESY correlations of H-1 with H-9/H-4 and H-3 with H-10 in **2** indicated the relative configurations of C-1, 3, 4 as (S*, R*, S*) (Figure 3 and Figure S16). The postulated dimeric mechanism for citrinin H1 had been proposed previously and was modified in this paper (Scheme 1) [5], which started with the reduction of 6-methoxymellein (**7**) to form intermediates **a** and **b** (keto-enol equilibrium), followed by dehydration and ring opening to obtain intermediate **c** and **11**, respectively. **11** was oxidized to the *para*-quinone-type intermediate **d**, followed by Diels-Alder reaction with intermediate **c** to form citrinin H1. Therefore, the relative configurations of C-7′ and C-8′ should be the same as that of the corresponding positions C-4 and C-3, respectively, and compound **2** was established as (1S*, 3R*, 4S*, 7′S*, 8′R*)-**2**. Similarly, NOESY correlations of H-1 with H-3/H-10 and H-9 with H-4 allowed the assignment of the relative configuration of **3** to be (1R*, 3R*, 4S*, 7′S*, 8′R*)-**3**. As the CD curve for citrinin H1 was not available in previous research, to unambiguously assign the absolute configuration of **2** and **3**, four possible isomers, (1*S*, 3*R*, 4*S*, 7′*S*, 8′*R*)-**2** and (1*R*, 3*R*, 4*S*, 7′*S*, 8′*R*)-**3** as well as their corresponding mirror images (1*R*, 3*S*, 4*R*, 7′*R*, 8′*S*)-**2** and (1*S*, 3*S*, 4*R*, 7′*R*, 8′*S*)-**3**, were found to satisfy the observed NOE constraints and were generated for TD-DFT calculation (Tables S3 and S4). The experimental ECD spectra of **2** and **3** were in good agreement with the calculated ECD spectrum of (1*S*, 3*R*, 4*S*, 7′*S*, 8′*R*)-**2** and (1*R*, 3*R*, 4*S*, 7′*S*, 8′*R*)-**3**, respectively (Figure 4). As the (1*R*, 3*R*, 4*S*, 7′*S*, 8′*R*)-**3** was previously described as citrinin H1 [5], compound **2** was assigned and named 1-*epi*-citrinin H1.

The known compounds (**3**–**13**) were identified as citrinin H1 (**3**) [5], penicitrinol A (**4**) [7], (3*S*,4*S*)-sclerotinin A (**5**) [8], stoloniferol B (**6**) [9], (3*R*)-6-methoxymellein (**7**) [10], (3*R*)-6-methoxy-7-chloromellein (**8**) [11], phenol A (**9**) [12], citrinin H2 (**10**) [4], (3*S)*-hydroxy-4-*epi*-isosclerone (**11**) [13], (3*R*,4*S*)-6,8-dihydroxy-1,1-dimethyl-3,4,5-trimethylisochroman (**12**) [14], and (3*S*)-(3′,5′-dihydroxy-2′-methylphenyl)-2-butanone (**13**) [15], by comparing their spectroscopic data with those reported in the literature.

The antibacterial and cytotoxic activity of compounds **1**–**13** were evaluated using strains of methicillin-resistant *Staphylococcus aureus* (MRSA) (ATCC 43300, CGMCC 1.12409), *Vibrio vulnificus* MCCC E1758, *Vibrio campbellii* MCCC E333, *Vibrio rotiferianus* MCCC E385, and A549 and HeLa cell lines (Table 2).

For strains of MRSA, compounds **1** and **2** showed similar activities in comparison to the positive control chloramphenicol with MIC values ranging from 7 to 8 μg/mL. For strains of *V. vulnificus*, compounds **1**–**13** showed weaker activities than the positive control erythromycin. Compounds **5** and **10** exhibited relatively stronger activities than the other compounds against *V. vulnificus* and *V. campbellii,* with MIC values ranging from 15 to 17 μg/mL, respectively.

For cytotoxicity, compounds **1**–**13** displayed weaker activities than the positive control doxorubicin. Compound **1** exhibited the most potent cytotoxic activities towards HeLa cells, with the IC_50_ value of 4.1 μM.

## 3. Materials and Methods

### 3.1. General Experimental Procedures

NMR spectra were recorded on a Bruker Avance 600 MHz spectrometer. HRESIMS data were obtained using a Xevo G2 Q-TOF mass spectrometer (Waters, Milford, MA, USA). CD spectra were measured on a J-715 spectropolarimeter (JASCO Corporation, Tokyo, Japan). Optical rotations were recorded on a Rudolph IV Autopol automatic polarimeter (Hackettstown, NJ, USA). The UV spectra were recorded on a UV-1800 spectrophotometer (Shimadzu, Kyoto, Japan). For column chromatography (CC), Sephadex LH-20 (GE Healthcare Bio-Science AB, Pittsburgh, PA, USA), silica gel (200–300 mesh, 300–400 mesh, Tsingtao Marine Chemical Co. Ltd., Tsingtao, China) and RP-C18 (ODS-A, 50 µm, YMC, Kyoto, Japan) were used. Preparative HPLC was run with a P3000 pump (CXTH, Beijing, China) and a UV3000 ultraviolet-visible detector (CXTH, Beijing, China), using a preparative RP-C18 column (5 µm, 20 mm × 250 mm, YMC, Kyoto, Japan). IR data were measured on a Bruker Tensor 27 spectrometer.

### 3.2. Fungal Material

The fungal strain was identified based on ITS sequence homology (99% similarity with *P. citrinum* strain SFC101144 with Genbank Accession No. MF185963.1 (max score 900, e value 0.0, query cover 100%)). The ITS1-5.8S-ITS2 rDNA sequence of the fungus has been submitted to GenBank with the accession number MK266987 (Text S1). The strain was deposited at the Third Institute of Oceanography, Ministry of Natural Resources, China.

### 3.3. Fermentation, Extraction, and Isolation

Strain NLG-S01-P1 was cultured on PDA plates at 28 °C for 3 days. Then, six plugs (5 mm diameter) were transferred to 12 Erlenmeyer flasks (1 L), each containing 500 mL PD medium in sterile conditions. Erlenmeyer flasks were shaken on a rotary shaker at 28 °C and 120 rpm for 3 days to form seed cultures (1 × 10^8^ spores/mL). Next, seed cultures (40 × 100 mL) were transferred to flasks (40 × 1 L) containing 70 g of rice, 0.1 g of corn flour, 0.3 g of peptone, 0.1 g of sodium glutamate, and 100 mL of naturally sourced and filtered seawater. After 28 days, the fermented broth was dried, smashed, and extracted with EtOAc. The organic solvent was evaporated under reduced pressure to afford the EtOAc extract (40 g), which was subjected to silica gel column chromatography using PE-acetone (9.8:0.2, 9.5:0.5, 9:1, 8.5:1.5, 8.0:2.0, 7.0:3.0, *V*:*V*) to yield five fractions, A–F. Fraction C was applied to Sephadex LH-20 (methanol) to yield four subfractions, C1–C4. Subfraction C2 was further purified by semi-preparative HPLC (70% methanol in H_2_O, flow rate 8 mL/min) to give compounds **10** (1.6 mg), **13** (1.0 mg), **1** (4.0 mg), **11** (14.4 mg), **5** (1.6 mg), and **6** (26.3 mg). Subfractions C3 and C4 were further separated by semi-preparative HPLC (70% methanol in H_2_O, flow rate 8 mL/min) to give compounds **7** (2.3 mg) and **4** (9.7 mg), respectively. Fraction D was applied to preparative HPLC (70% methanol in H_2_O, flow rate 12 mL/min) to yield compounds **8** (2.2 mg), **12** (1.1 mg), **3** (1.2 mg), and **2** (1.0 mg). Fraction E was applied to Sephadex LH-20 (methanol) to yield two subfractions, E1 and E2. Subfraction E1 was further purified by semi-preparative HPLC (85% methanol in H_2_O, flow rate 8 mL/min) to give compound **9** (2.1 mg).

Penicitol D (**1**): yellow amorphous solid; [α]${}_{D}^{25}$ – 6.3 (*c* 0.09, MeOH); UV λ_max_ (methanol) nm (log *ε*): 216 (4.62), 275 (4.23), 306 (3.83); ^1^H NMR and ^13^C NMR data are shown in Table 1; HR-ESI-MS: *m*/*z* 427.1747 [M − H]^−^ (Calcd. for 427.1757, C_24_H_27_O_7_, ∆—2.3 ppm); IR (KBr) *v*_max_ 3227, 2934, 1644, 1474, 1365, 1216, 1178, 1079, 1018, 965 cm^−1^.

1-*epi*-citrinin H1 (**2**): red amorphous solid; [α]${}_{D}^{25}$ – 22.3 (*c* 0.03, MeOH); UV λ_max_ (methanol) nm (log *ε*): 207 (4.59), 270 (4.17); ^1^H NMR and ^13^C NMR data are shown in Table 1; HR-ESI-MS: *m*/*z* 449.1576 [M + Na]^+^ (Calcd. for 449.1576, C_24_H_26_O_7_Na, ∆—0.0 ppm); IR (KBr) *v*_max_ 3403, 2975, 2931, 1720, 1652, 1595, 1493, 1455, 1381, 1309, 1257, 1185, 1108, 1012, 962 cm^−1^.

### 3.4. ECD Calculation

Conformational searches were carried out by means of a Merck Molecular Force Field (MMFF). A restricted number of low-energy conformations were obtained falling in an energy window with the threshold of 5 kcal/mol, and then conformers were initially optimized at B3LYP/6-31G(d) level in MeOH using the conductor-like polarizable continuum model (CPCM). The theoretical calculations were carried out using Gaussian 09 (Wallingford, CT, USA). The optimized-conformers with Boltzmann-based population lower than 1% were filtered out, and the remaining chosen for ECD calculation were conducted using TD-DFT at wB97XD/def2-SVP [16]. Rotatory strengths for a total of 30 excited states were calculated. The Boltzmann-averaged ECD spectrum was obtained with the aid of Multiwfn 3.6 software. Electronic transitions were expanded as Gaussian curves, with a full width at half maximum (FWHM) for each peak set to 0.3 eV, which gives a good fitting with the experimental width of the bands [17].

### 3.5. Antibacterial Assay

Antibacterial activities against MRSA (ATCC 43300, CGMCC 1.12409), *V. vulnificus* (MCCC E1758), *V. campbellii* (MCCC E333), and *V. rotiferianus* (MCCC E385) were tested by continuous dilution in 96-well plates using resazurin as a surrogate indicator. The MIC value was determined to be the lowest concentration that did not induce the color change by observing the blue-to-pink color change [18,19,20].

### 3.6. Cytotoxicity Assay

Cytotoxic activities of compounds **1**–**13** were evaluated using A549 (adenocarcinomic human alveolar basal epithelial cell) and HeLa (cervical cancer cell) cells by Cell Counting Kit-8 (CCK-8) (DOJINDO) assay at 48 h post-treatment with doxorubicin as a positive control, following the manufacturer’s instructions [21]. The cell lines were purchased from the cell bank of Chinese Academy of Sciences, Shanghai, China.

## 4. Conclusions

In the current research, we have isolated four citrinin dimer and nine monomer derivatives, including two novel dimers, penicitol D (**1**) and 1-*epi*-citrinin H1 (**2**). The absolute configuration of **1**–**2** was elucidated using the TD-DFT ECD method and a plausible biogenetic pathway for **2**–**4** was proposed. Compounds **1** and **2** showed similar anti-MRSA activities in comparison to chloramphenicol, and compound **1** displayed the most potent cytotoxic activity towards HeLa cells, with an IC_50_ below 5 μM. Both 1-*epi*-citrinin H1 (**2**)—as an epimer of citrinin H1—and the anti-vibiro activities of compounds **1**–**13** are reported here for the first time. Penicitol D (**1**) is the second citrinin dimer with a single oxygen-bridging center with a similar structure skeleton to the previously reported penicitol B [22]. The previously unreported compounds in the current study enrich the structural diversity of citrinin dimers. 

## Figures and Tables

**Table 1:** ^1^H NMR data (600 MHz) and ^13^C NMR data (150 MHz) for compounds **1** and **2** (acetone-*d6*) (Figures S1, S2, S9 and S10).

Position	1		2	
	δ_C_, Type	δ_H_, Mult. (*J* in Hz)	δ_C_, Type	δ_H_, Mult. (*J* in Hz)
1	58.9, CH_2_	4.54, d (15.4)	61.3, CH	5.29, s
1	58.9, CH_2_	4.61, d (15.4)		
3	73.8, CH	3.85, qd (6.54, 2.3)	79.6, CH	4.03, qd (6.5, 4.6)
4	35.1, CH	2.64, qd (6.87, 2.3)	37.7, CH	2.97, qd (7.4, 4.6)
4a	137.9, C		140.6, C	
5	112.7, C		120.1, C	
6	154.2, C		156.1, C	
7	99.7, CH	6.32, s	99.6, CH	6.61, s
8	150.8, C		144.9, C	
8a	111.8, C		111.1, C	
9	17.4, CH_3_	1.19, d (6.5)	14.8, CH_3_	1.32, d (6.5)
10	19.9, CH_3_	1.21, d (6.9)	22.0, CH_3_	1.26, d (7.4)
11	9.5, CH_3_	2.06, s	10.0, CH_3_	2.17, s
1′	170.2, C		179.8, C	
2′			143.0, C	
3′	102.3, C		142.3, C	
4′	36, CH_2_	3.09, d (18.0)	184.8, C	
4′	36, CH_2_	3.20, d (18.0)		
4′a	134.5, C			
5′	113.4, C		115.2, C	
6′	159.8, C		149.0, C	
7′	100.5, C		40.3, CH	3.22, qd (7.3, 2.4)
8′	160, C		72.3, CH	5.48, qd (6.1,2.4)
8′a	108.7, C			
9′	27.4, CH_3_	1.71, s	20.5, CH_3_	1.34, d (7.3)
10′	7.3, CH_3_	2.13, s	18.3, CH_3_	1.39, d (6.1)
11′	10.3, CH_3_	2.13, s	11.4, CH_3_	2.14, s
12′			160.5, CH	7.97, s
OH-6				8.82, s
OH-8′		11.82, s		
OH-8		7.98, s		
OH-6′		7.82, s		

**Table 2:** Antimicrobial and cytotoxic activities of compounds **1**–**13**. Data are expressed as mean ± SD values of three independent experiments, each made in triplicate.

Compounds	MIC (μg/mL)					IC_50_ (μM)	
	MRSA 1	MRSA 2	VV	VC	VR	A549	HeLa
**1**	7.8 ± 0.8	7.6 ± 0.5	NA	30.1 ± 1.2	NA	23.2 ± 1.2	4.1 ± 0.8
**2**	7.3 ± 0.8	7.8 ± 0.9	NA	32.7± 1.9	NA	45.2 ± 0.9	42.3 ± 0.6
**3**	15.3 ± 0.6	15.5 ± 0.8	NA	32.3 ± 0.3	NA	46.3 ± 0.7	35.6 ± 0.9
**4**	16.3 ± 0.5	32.2 ± 0.3	NA	NA	NA	23.1 ± 0.9	17.7 ± 0.3
**5**	15.2 ± 0.4	16.1 ± 0.3	16.6 ± 0.4	15.3 ± 0.4	32.3 ± 0.3	40.0 ± 0.3	42.2 ± 0.5
**6**	NA	NA	NA	NA	NA	NA	NA
**7**	NA	NA	32.4 ± 0.5	NA	33.3 ± 0.2	NA	NA
**8**	NA	NA	NA	NA	NA	25.1 ± 1.0	27.7 ± 0.2
**9**	NA	NA	NA	NA	33.1 ± 0.7	33.1 ± 0.9	16.7 ± 0.1
**10**	NA	NA	15.7 ± 0.6	15.6 ± 0.5	32.3 ± 0.3	42.0 ± 0.3	22.2 ± 0.4
**11**	33.6 ± 0.2	32.1 ± 0.1	NA	NA	NA	37.7 ± 0.3	NA
**12**	32.1 ± 0.4	32.4 ± 0.1	32.1 ± 0.5	NA	NA	NA	NA
**13**	NA	NA	NA	NA	32.7 ± 0.2	NA	NA
erythromycin	NT	NT	2.0 ± 0.0	7.7 ± 0.2	3.9 ± 0.1	NT	NT
chloramphenicol	7.6 ± 0.2	7.5 ± 0.1	NT	NT	NT	NT	NT
doxorubicin	NT	NT	NT	NT	NT	0.1 ± 0.0	0.5 ± 0.1

MRSA 1: methicillin-resistant *S. aureus* ATCC 43300; MRSA 2: methicillin-resistant *S. aureus* CGMCC 1.12409; VV: *V. vulnificus* MCCC E1758; VC: *V. campbellii* MCCC E333; VR: *V. rotiferianus* MCCC E385; NA: no activity at the concentration of 50 μg/mL (antibacterial) or 50 μM (cytotoxic); NT: not tested.

## Supplementary Materials

NMR spectra for compounds **1**–**2** as well as computational data for (3*S*, 4*R*, 3′*R*)-**1**, (3*S*, 4*R*, 3′*S*)-**1**, (1*S*, 3*R*, 4*S*, 7′*S*, 8′*R*)-**2**, and (1*R*, 3*R*, 4*S*, 7′*S*, 8′*R*)-**3** are available online at http://www.mdpi.com/1660-3397/17/1/46/s1, Figures S1–S16: NMR data of compounds **1**–**2**; Tables S1–S4: Gibbs free energies and equilibrium populations of the calculated conformers; Text S1: ITS sequence of strain NLG-S01-P1.
