# Supplementary material for: Citrinin Monomer and Dimer Derivatives with Antibacterial and Cytotoxic Activities Isolated from the Deep Sea-Derived Fungus Penicillium citrinum NLG-S01-P1
Source: Mar Drugs. 2019 Jan 10;17(1):46. doi: 10.3390/md17010046 (PMC6357177; doi:10.3390/md17010046)
Supplement: Supplementary file 1 [file marinedrugs-17-00046-s001.pdf]

Supplementary Files

Citrinin Monomer and Dimer Derivatives with Antibacterial and Cytotoxic Activities Isolated from the Deep Sea-Derived Fungus *Penicillium citrinum* NLG-S01-P1

Weiyi Wang [1,*], Yanyan Liao [2], Beibei Zhang [1], Maolin Gao [1], Wenqian Ke [1], Fang Li [1] and Zongze Shao [1]

[1] Key Laboratory of Marine Genetic Resources, State Key Laboratory Breeding Base of Marine Genetic Resources, Fujian Key Laboratory of Marine Genetic Resources, Fujian Collaborative Innovation Centre for Exploitation and Utilization of Marine Biological Resources, Third Institute of Oceanography, Ministry of Natural Resources, Xiamen 361005, China; zbb953372968@163.com (B.Z.); g17785208284@163.com (M.G.); 15260587285@163.com (W.K.); lifang@tio.org.cn (F.L.); shaozongze@tio.org.cn (Z.S.)

[2] Key Laboratory of Urban Environment and Health, Institute of Urban Environment, Chinese Academy of Sciences, Xiamen 361021, China; yyliao@iue.ac.cn

* Correspondence: wywang@tio.org.cn; Tel.: +86-592-219-5518

Content

Figure S1. ^{1}H NMR spectrum of compound **1** 2

Figure S2. ^{13}C NMR spectrum of compound **1** 2

Figure S3. HSQC spectrum of compound **1** 2

Figure S4. HMBC spectrum of compound **1** 3

Figure S5. HRESIMS spectrum of compound **1** 3

Figure S6. ^{13}C/DEPT spectrum of compound **1** 3

Figure S7. ^{1}H-^{1}H COSY spectrum of compound **1** 4

Figure S8. NOESY spectrum of compound **1** 4

Figure S9. ^{1}H NMR spectrum of compound **2** 5

Figure S10. ^{13}C NMR spectrum of compound **2** 5

Figure S11. HSQC spectrum of compound **2** 5

Figure S12. HMBC spectrum of compound **2** 6

Figure S13. HRESIMS spectrum of compound **2** 6

Figure S14. ^{13}C/DEPT spectrum of compound **2** 6

Figure S15. ^{1}H-^{1}H COSY spectrum of compound **2** 7

Figure S16. NOESY spectrum of compound **2** 7

Table S1. Gibbs free energies ^{a} and equilibrium populations ^{b} of low-energy conformers of (3*S*, 4*R*, 3′*R*)-**1** in methanol 8

Table S2. Gibbs free energies ^{a} and equilibrium populations ^{b} of low-energy conformers of (3*S*, 4*R*, 3′*S*)-**1** in methanol 8

Table S3. Gibbs free energies ^{a} and equilibrium populations ^{b} of low-energy conformers of (1*S*, 3*R*, 4*S*, 7′*S*, 8′*R*)-**2** in methanol 9

Table S4. Gibbs free energies ^{a} and equilibrium populations ^{b} of low-energy conformers of (1*R*, 3*R*, 4*S*, 7′*S*, 8′*R*)-**3** in methanol 10

Text S1. ITS1-5.8S-ITS2 rDNA sequence of strain NLG-S01-P1 11

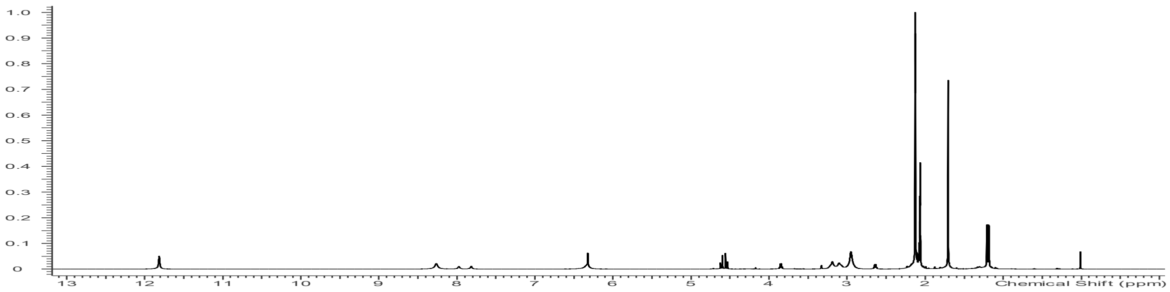

Figure S1. ^{1}H NMR spectrum of compound **1**

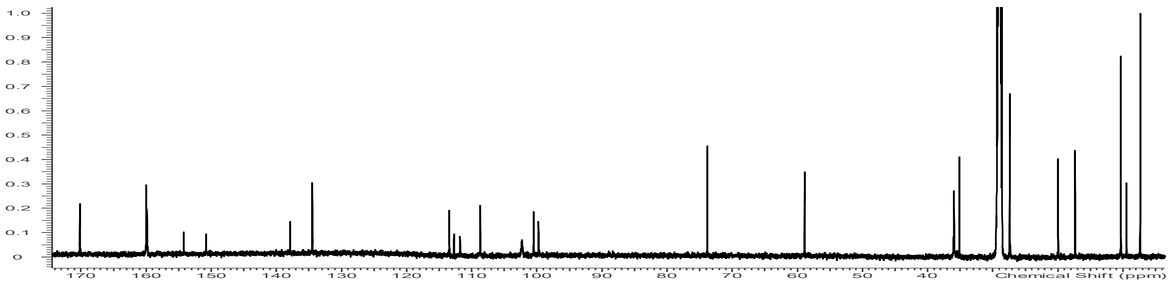

Figure S2. ^{13}C NMR spectrum of compound **1**

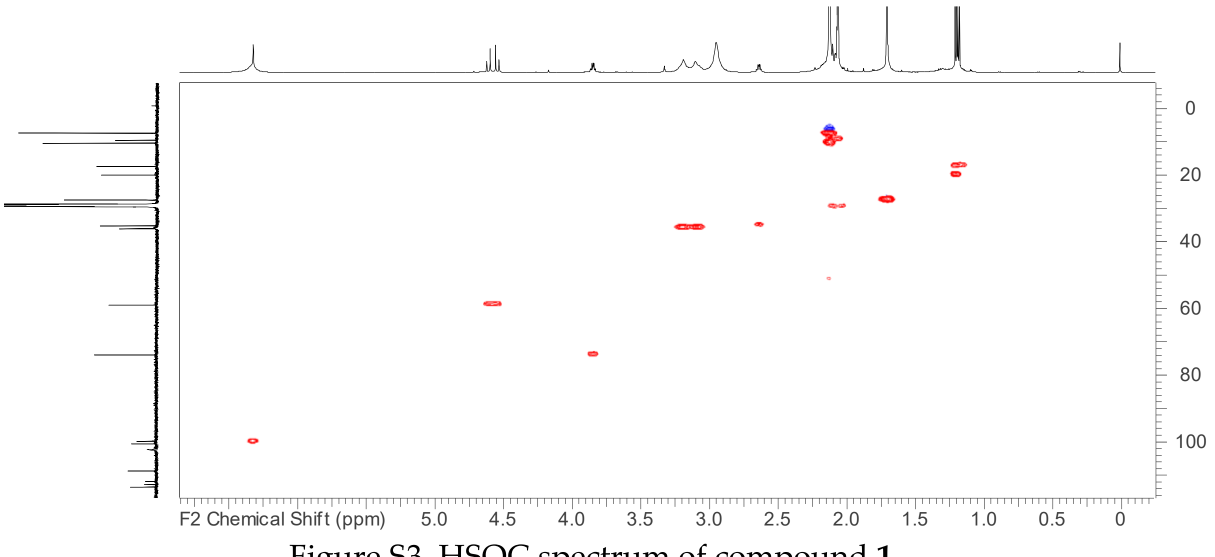

Figure S3. HSQC spectrum of compound **1**

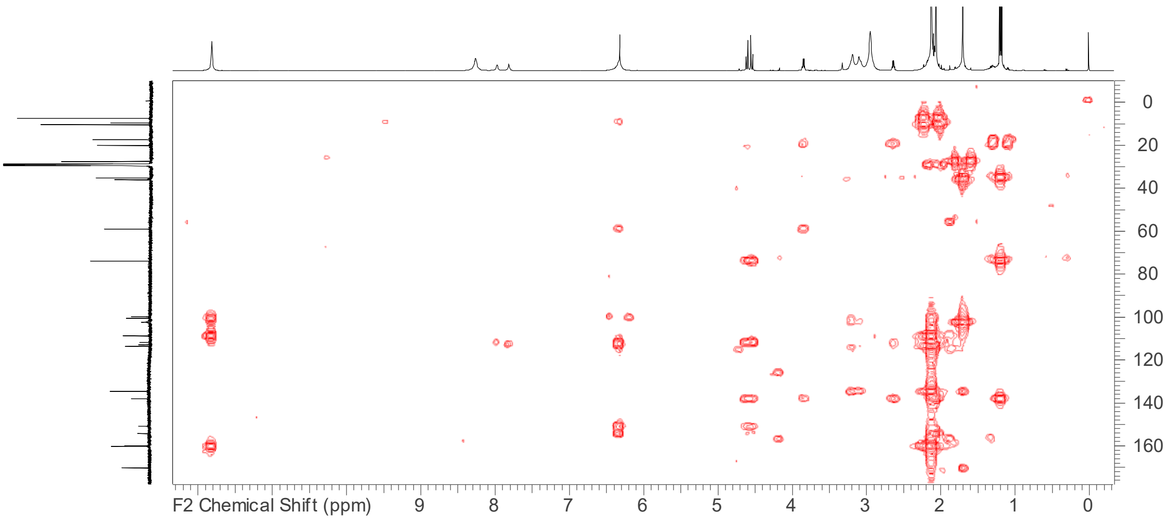

Figure S4. HMBC spectrum of compound **1**

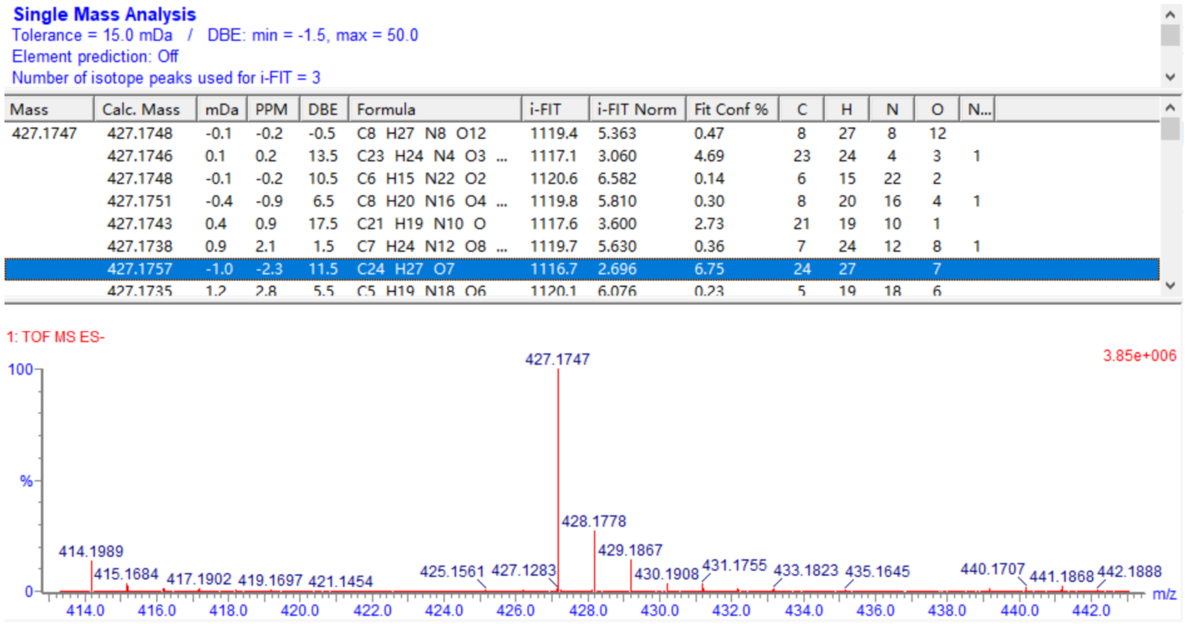

Mass	Calc. Mass	mDa	PPM	DBE	Formula	i-FIT	i-FIT Norm	Fit Conf %	C	H	N	O	N...
427.1747	427.1748	-0.1	-0.2	-0.5	C8 H27 N8 O12	1119.4	5.363	0.47	8	27	8	12	
	427.1746	0.1	0.2	13.5	C23 H24 N4 O3 ...	1117.1	3.060	4.69	23	24	4	3	1
	427.1748	-0.1	-0.2	10.5	C6 H15 N22 O2	1120.6	6.582	0.14	6	15	22	2	
	427.1751	-0.4	-0.9	6.5	C8 H20 N16 O4 ...	1119.8	5.810	0.30	8	20	16	4	1
	427.1743	0.4	0.9	17.5	C21 H19 N10 O	1117.6	3.600	2.73	21	19	10	1	
	427.1738	0.9	2.1	1.5	C7 H24 N12 O8 ...	1119.7	5.630	0.36	7	24	12	8	1
	427.1757	-1.0	-2.3	11.5	C24 H27 O7	1116.7	2.696	6.75	24	27		7	
	427.1735	1.2	2.8	5.5	C5 H19 N18 O6	1120.1	6.076	0.23	5	19	18	6	

Figure S5. HRESIMS spectrum of compound **1**

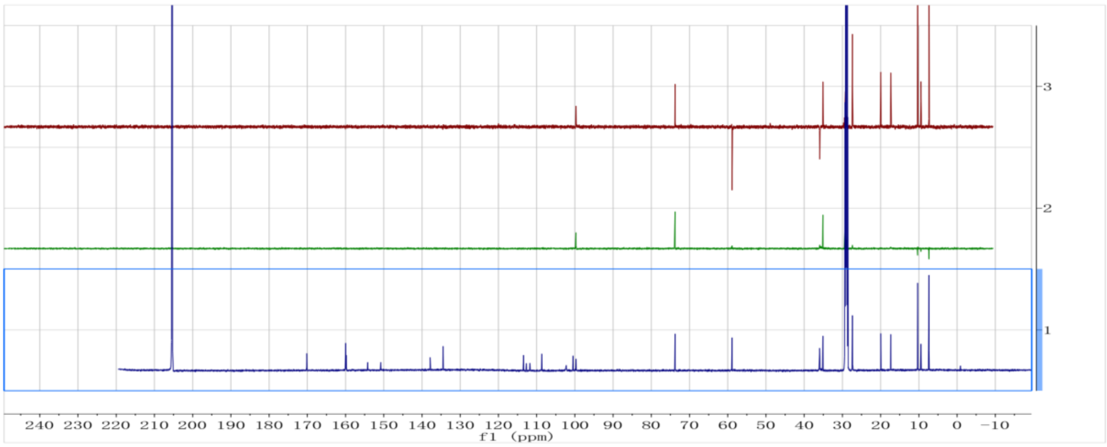

Figure S6. ^{13}C/DEPT spectrum of compound **1**

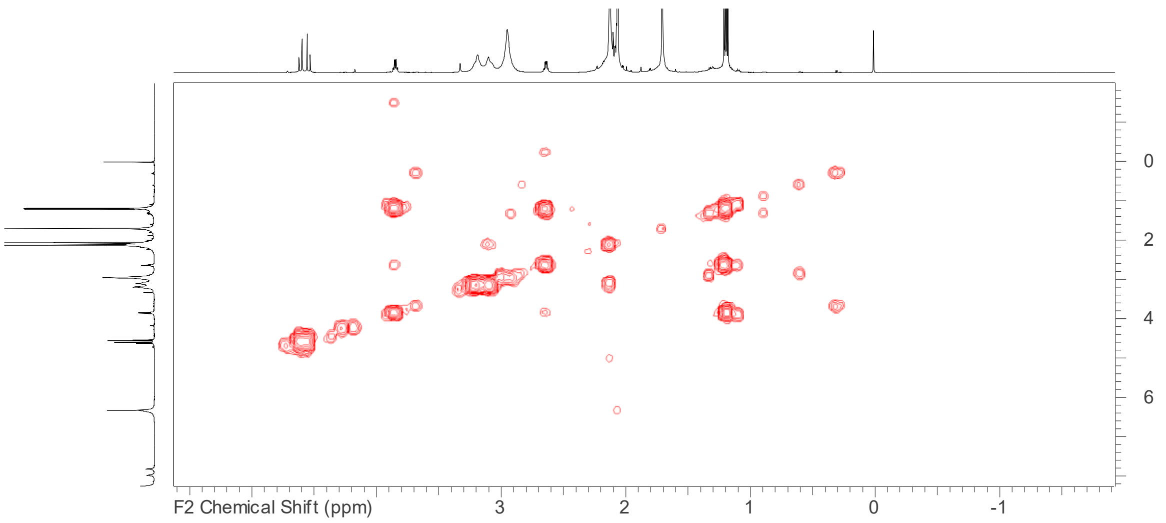

Figure S7. ^{1}H-^{1}H COSY spectrum of compound **1**

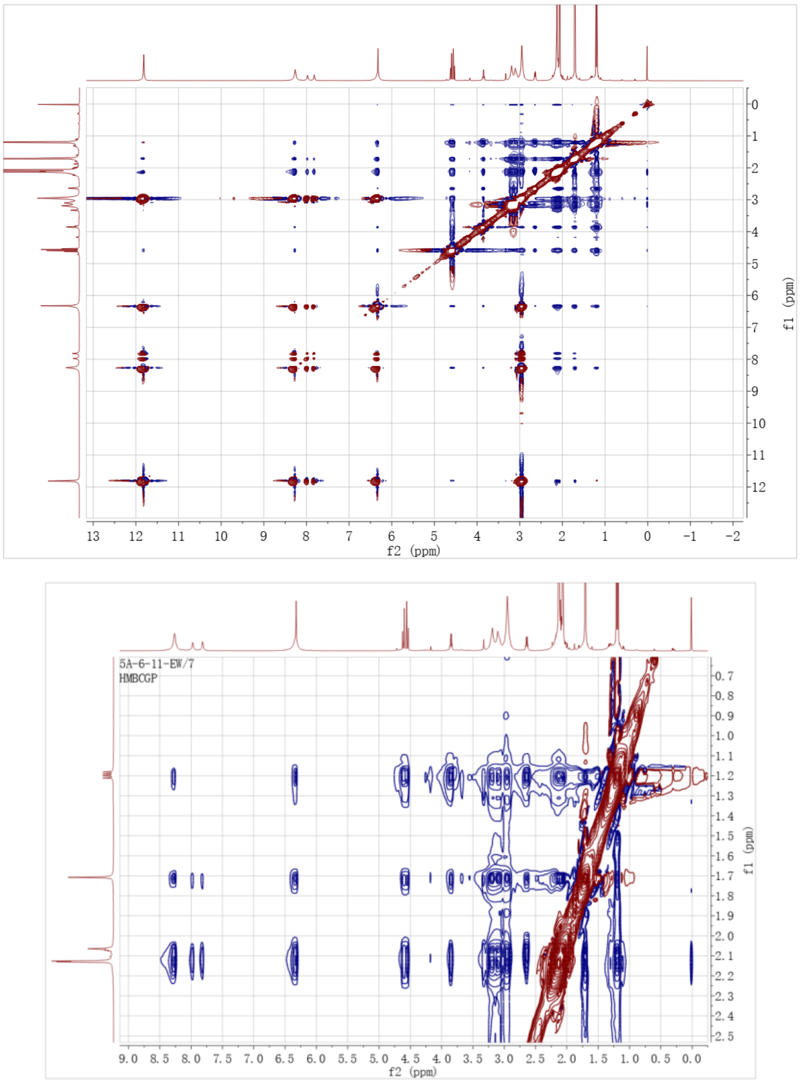

Figure S8. NOESY spectrum of compound **1**

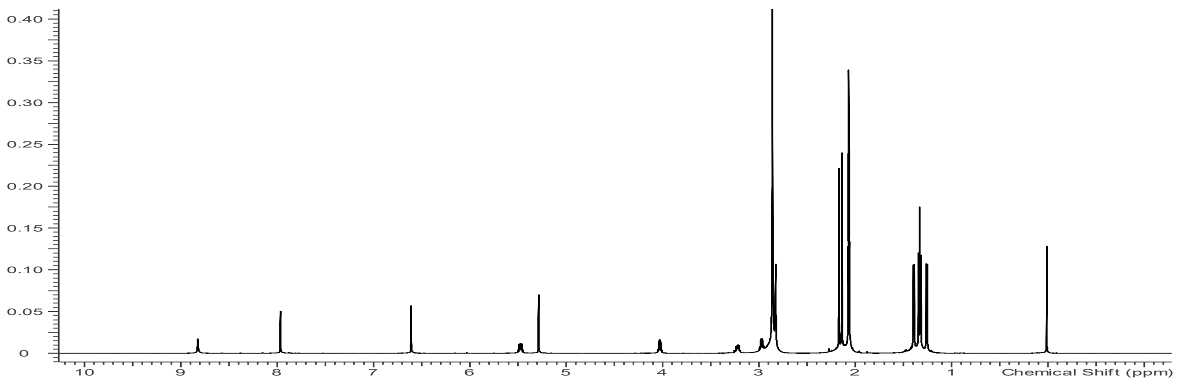

Figure S9. ^{1}H NMR spectrum of compound **2**

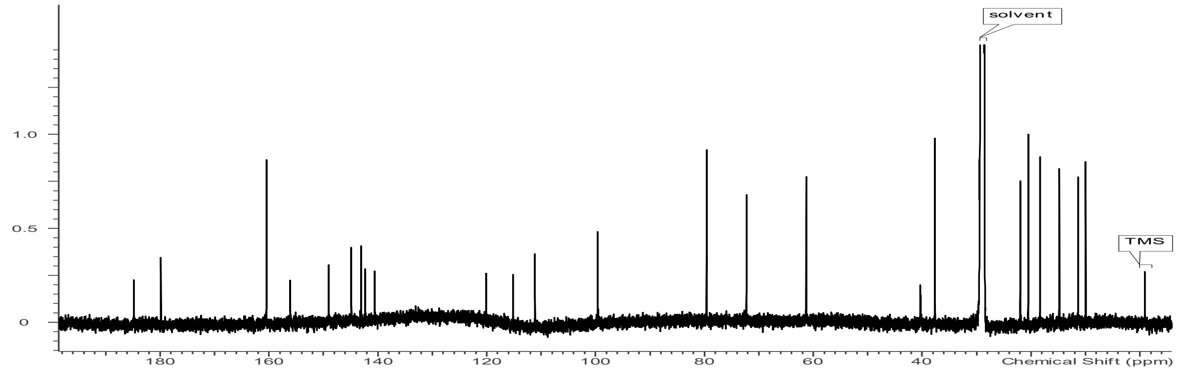

Figure S10. ^{13}C NMR spectrum of compound **2**

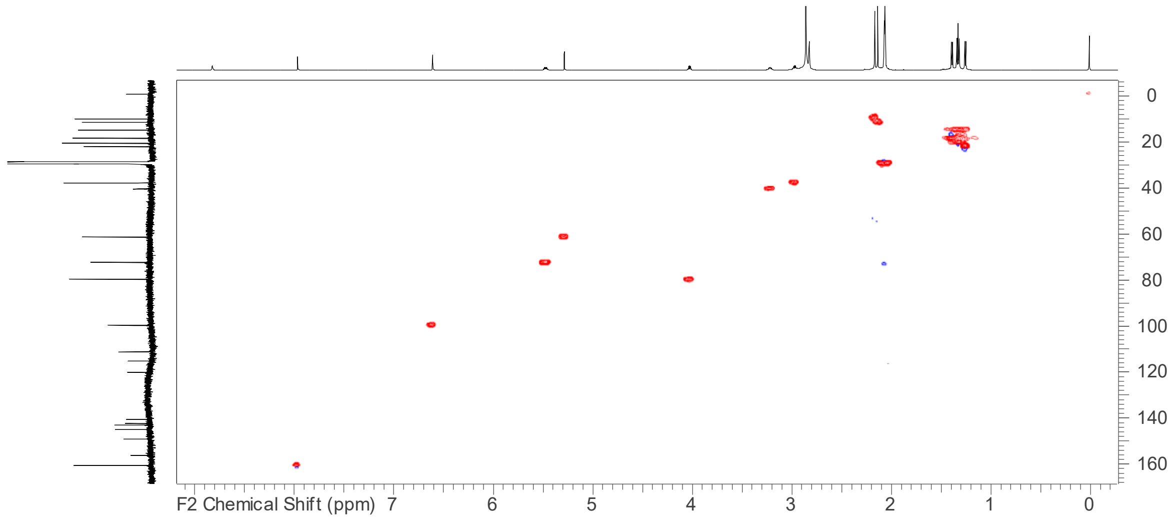

Figure S11. HSQC spectrum of compound **2**

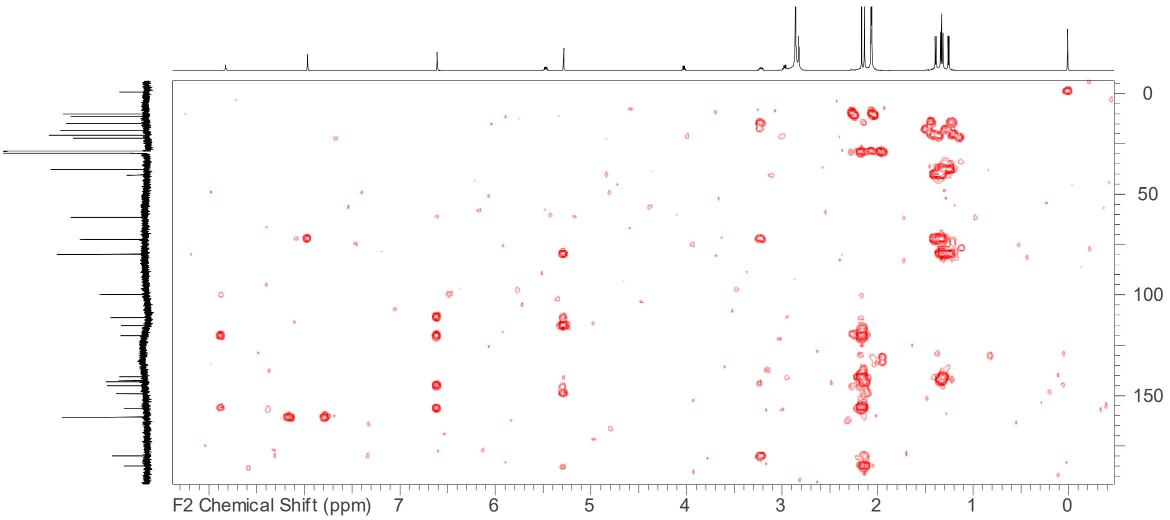

Figure S12. HMBC spectrum of compound **2**

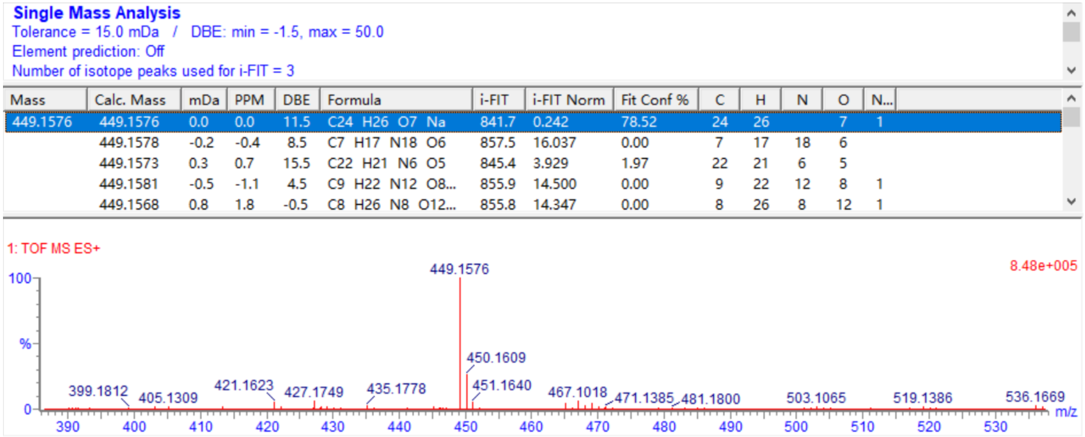

Mass	Calc. Mass	mDa	PPM	DBE	Formula	i-FIT	i-FIT Norm	Fit Conf %	C	H	N	O	N...
449.1576	449.1576	0.0	0.0	11.5	C24 H26 O7 Na	841.7	0.242	78.52	24	26		7	1
	449.1578	-0.2	-0.4	8.5	C7 H17 N18 O6	857.5	16.037	0.00	7	17	18	6	
	449.1573	0.3	0.7	15.5	C22 H21 N6 O5	845.4	3.929	1.97	22	21	6	5	
	449.1581	-0.5	-1.1	4.5	C9 H22 N12 O8...	855.9	14.500	0.00	9	22	12	8	1
	449.1568	0.8	1.8	-0.5	C8 H26 N8 O12...	855.8	14.347	0.00	8	26	8	12	1

Figure S13. HRESIMS spectrum of compound **2**

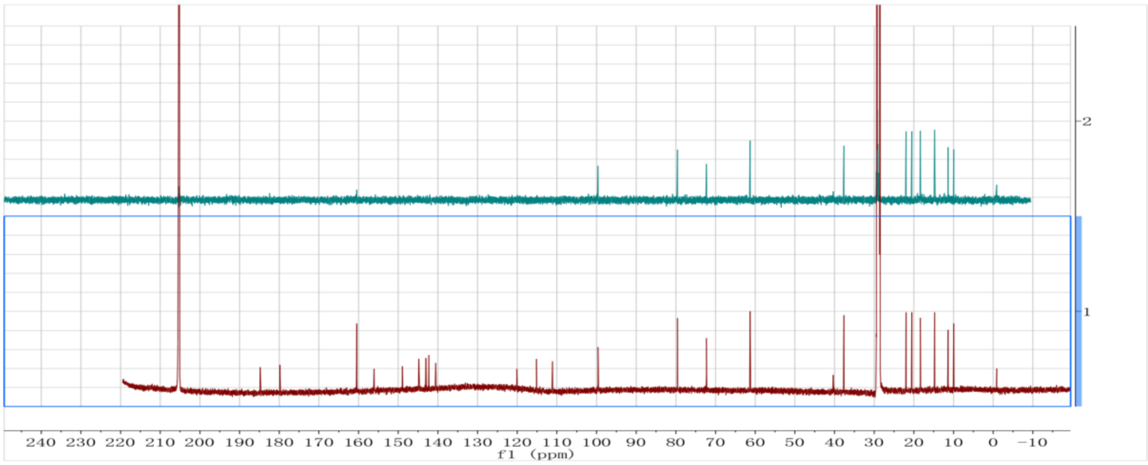

Figure S14. ^{13}C/DEPT spectrum of compound **2**

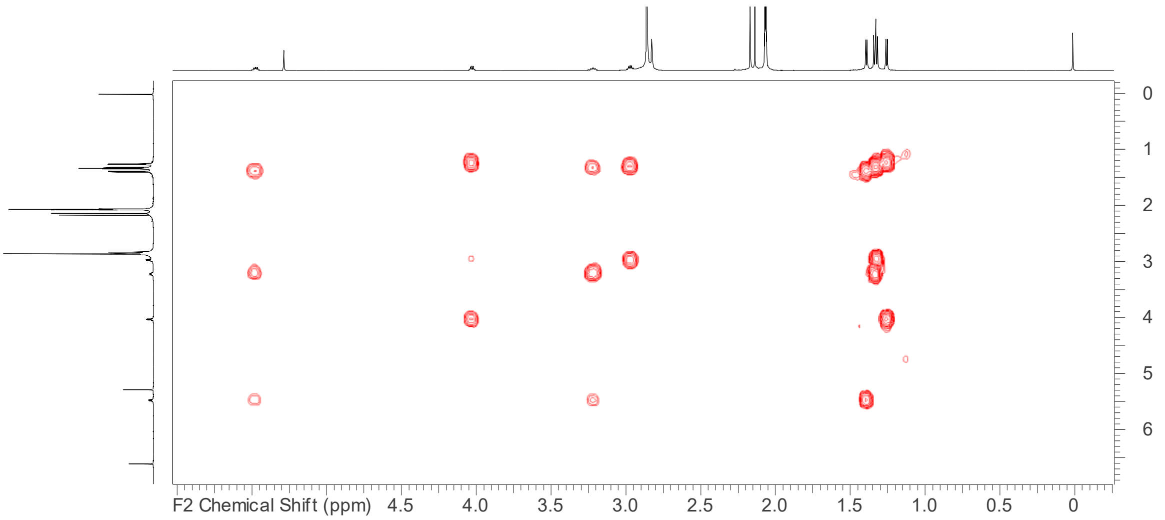

Figure S15. ^1H-^1H COSY spectrum of compound **2**

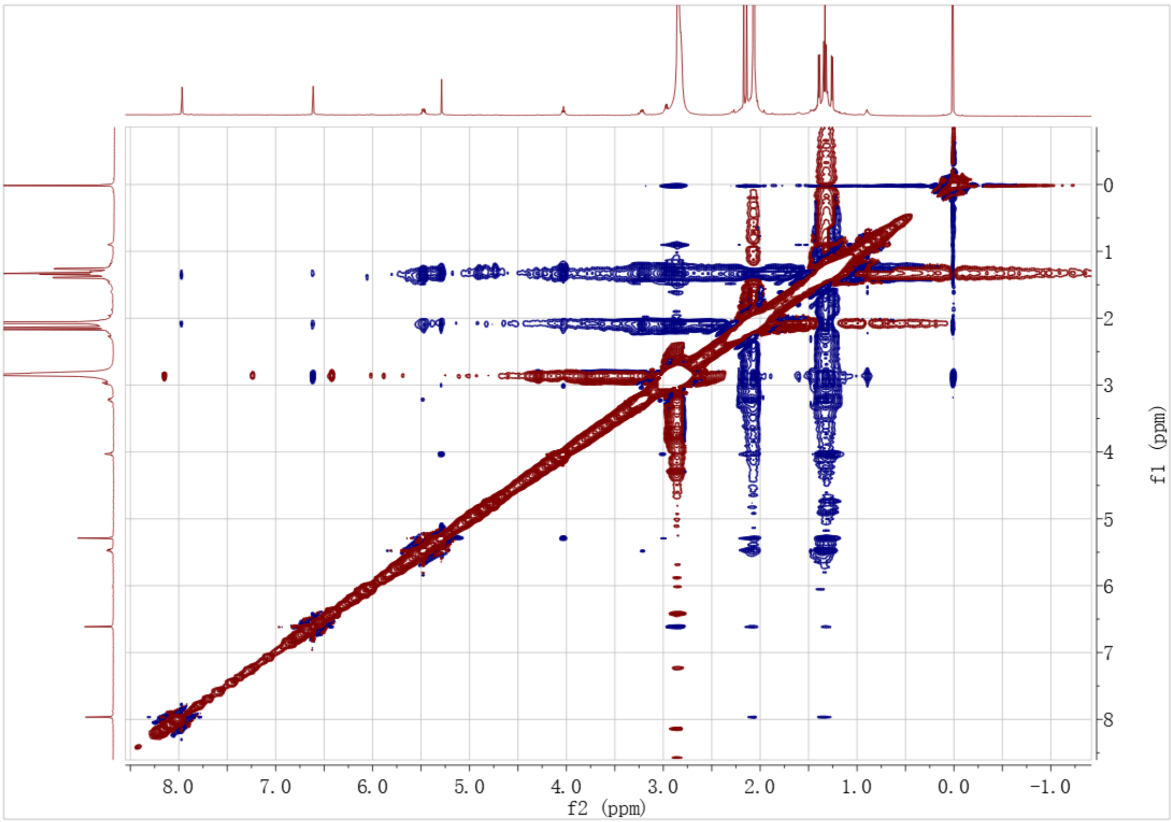

Figure S16. NOESY spectrum of compound **2**

Table S1. Gibbs free energies [a] and equilibrium populations [b] of low-energy conformers of (3*S*, 4*R*, 3′*R*)-**1**

configuration	conformer	structure	△G (kcal/mol)	Percent
(3*S*, 4*R*, 3′*R*)-**1**	a	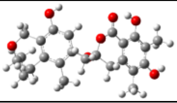	0.00000	29.29%
	b	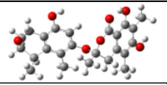	0.00188	29.20%
	c	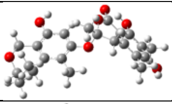	0.24221	19.46%
	d	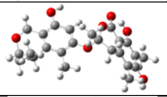	0.24472	19.38%
	e	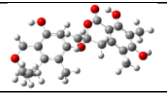	1.41815	2.67%

[a] **B3LYP/6-31G(d), in kcal/mol.** [b] **From ΔG values at 298.15 K.**

Table S2. Gibbs free energies [a] and equilibrium populations [b] of low-energy conformers of (3*S*, 4*R*, 3′*S*)-**1**

configuration	conformer	structure	△G (kcal/mol)	Percent
(3*S*, 4*R*, 3′*S*)-**1**	a	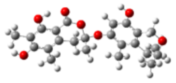	0.00000	68.58%
	b	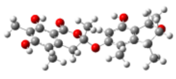	0.72037	20.31%
	c	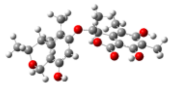	1.28449	7.83%
	d	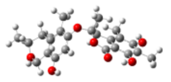	1.80155	3.27%

[a] **B3LYP/6-31G(d), in kcal/mol.** [b] **From ΔG values at 298.15 K.**

Table S3. Gibbs free energies [a] and equilibrium populations [b] of low-energy conformers of (1*S*, 3*R*, 4*S*, 7′*S*, 8′*R*)-**2**

configuration	conformer	structure	△G (kcal/mol)	Percent
(1*S*, 3*R*, 4*S*, 7′*S*, 8′*R*)-2	a	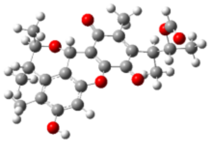	0.00000	58.25%
	b	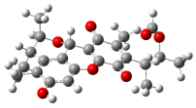	0.72727	17.06%
	c	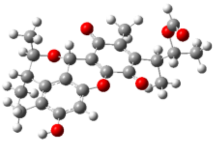	0.86783	13.45%
	d	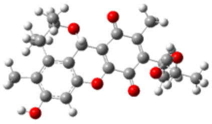	1.50851	4.56%
	e	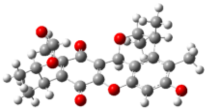	1.69174	3.35%
	f	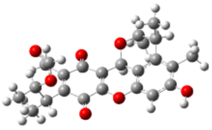	1.69362	3.33%

[a] B3LYP/6-31G(d), in kcal/mol. [b] From ΔG values at 298.15 K.

Table S4. Gibbs free energies [a] and equilibrium populations [b] of low-energy conformers of (1*R*, 3*R*, 4*S*, 7′*S*, 8′*R*)-**3**

configuration	conformer	structure	△G (kcal/mol)	Percent
(1*R*, 3*R*, 4*S*, 7′*S*, 8′*R*)-3	a	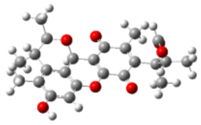	0.00000	48.79%
	b	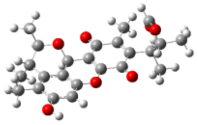	0.78312	13.00%
	c	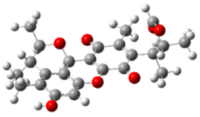	0.80696	12.49%
	d	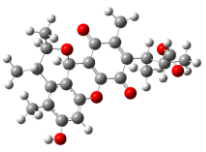	0.80885	12.45%
	e	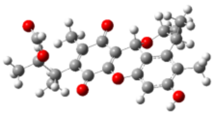	1.54930	3.56%
	f	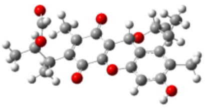	1.55494	3.53%
	g	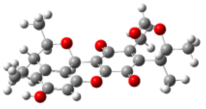	1.85740	2.12%
	h	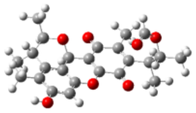	1.85928	2.11%
	i	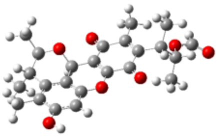	1.90572	1.95%

[a] B3LYP/6-31G(d), in kcal/mol. [b] From ΔG values at 298.15 K.

Text S1. ITS1-5.8S-ITS2 rDNA sequence of strain NLG-S01-P1

```
LOCUS       Seq1                    487 bp    DNA     linear   PLN 05-DEC-2018
DEFINITION  Penicillium citrinum NLG-S01-P1 18S ribosomal RNA gene, partial
            sequence; internal transcribed spacer 1, 5.8S ribosomal RNA gene,
            and internal transcribed spacer 2, complete sequence; and 28S
            ribosomal RNA gene, partial sequence.
ACCESSION   Seq1
VERSION
KEYWORDS    .
SOURCE      Penicillium citrinum
  ORGANISM  Penicillium citrinum
            Eukaryota; Fungi; Dikarya; Ascomycota; Pezizomycotina;
            Eurotiomycetes; Eurotiomycetidae; Eurotiales; Aspergillaceae;
            Penicillium.
REFERENCE   1  (bases 1 to 487)
  AUTHORS   Wang,W.
  TITLE     Citrinin Monomer and Dimer Derivatives with Antibacterial and
            Cytotoxic Activities Isolated from the Deep Sea Derived Fungus
            Penicillium citrinum NLG-S01-P1
  JOURNAL   unpublished
REFERENCE   2  (bases 1 to 487)
  AUTHORS   Wang,W.
  TITLE     Direct Submission
  JOURNAL   Submitted (05-DEC-2018) Key Laboratory of Marine Biogenetic
            Resources, Third Institute of Oceanography, State Oceanic
            Administration, 178 Daxue Road, Xiamen, Fujian 361005, China
COMMENT     Bankit Comment: ALT EMAIL:wywang_cas@163.com
            Bankit Comment: TOTAL # OF SEQS:1

            ##Assembly-Data-START##
            Sequencing Technology :: Sanger dideoxy sequencing
            ##Assembly-Data-END##
FEATURES             Location/Qualifiers
     source          1..487
                     /organism="Penicillium citrinum"
                     /mol_type="genomic DNA"
                     /isolation_source="seawater"
                     /bio_material="Penicillium citrinum"
                     /db_xref="taxon:5077"
                     /collected_by="Yanping Hou"
                     /identified_by="Weiyi Wang"
     misc_RNA        1..487
                     /note="contains 18S ribosomal RNA gene, partial sequence;
                     internal transcribed spacer 1, 5.8S ribosomal RNA gene,
                     and internal transcribed spacer 2, complete sequence; and
                     28S ribosomal RNA gene, partial sequence"
BASE COUNT      97 a    138 c    161 g    91 t
ORIGIN
        1 aattaaaggt tgggggtcgg ctggcgccgg ccgggcctac tagagcgggt gacgaagccc
```

61 catacgctcg aggaccggac gcggtgccgc cgctgccttt cgggcccgtc cccccggcgg
121 gggggacggg gcccaacaca caagccgggc ttgagggcag caatgacgct cggacaggca
181 tgccctccgg aataccagag ggcgcaatgt gcgttcaaag actcgatgat tcactgaatt
241 ctgcaattca cattagttat cgcatttcgc tgcgttcttc atcgatgccg gaaccaagag
301 atccgttgtt gaaagtttta actaatttcg ttataggtct cagactgcaa cttcagacag
361 cgttcagggg ggccgtcggc gggcgcgggg cccgccgagg caacataggt tcgggcaaca
421 cggggggggag gttgggcccc gaggggcccg cactcggtaa tgatccttcc gcaggttcac
481 ctacgga
//
